# Identification of quantitative trait loci and associated candidate genes for pregnancy success in Angus–Brahman crossbred heifers

**DOI:** 10.1186/s40104-023-00940-2

**Published:** 2023-11-07

**Authors:** Quinn A. Hoorn, Gabriel A. Zayas, Eduardo E. Rodriguez, Laura M. Jensen, Raluca G. Mateescu, Peter J. Hansen

**Affiliations:** 1 Department of Animal Sciences, Donald Henry Barron Reproductive and Perinatal Biology Research Program, and the Genetics Institute, University of Florida, Gainesville, FL USA; 2 Present address: School of Applied Systems Biology, La Trobe University, Bundoora, VIC 3083 Australia

**Keywords:** Beef cattle, Fertility, GWAS, QTL

## Abstract

**Background:**

In beef cattle, more than 50% of the energy input to produce a unit of beef is consumed by the female that produced the calf. Development of genomic tools to identify females with high genetic merit for reproductive function could increase the profitability and sustainability of beef production.

**Results:**

Genome-wide association studies (GWAS) were performed using a single-step genomic best linear unbiased prediction approach on pregnancy outcome traits from a population of Angus–Brahman crossbred heifers. Furthermore, a validation GWAS was performed using data from another farm. Heifers were genotyped with the Bovine GGP F250 array that contains 221,077 SNPs. In the discovery population, heifers were bred in winter breeding seasons involving a single round of timed artificial insemination (AI) followed by natural mating for 3 months. Two phenotypes were analyzed: pregnancy outcome to first-service AI (PAI; *n* = 1,481) and pregnancy status at the end of the breeding season (PEBS; *n* = 1,725). The heritability was estimated as 0.149 and 0.122 for PAI and PEBS, respectively. In the PAI model, one quantitative trait locus (QTL), located between 52.3 and 52.5 Mb on BTA7, explained about 3% of the genetic variation, in a region containing a cluster of γ-protocadherin genes and *SLC25A2*. Other QTLs explaining between 0.5% and 1% of the genetic variation were found on BTA12 and 25. In the PEBS model, a large QTL on BTA7 was synonymous with the QTL for PAI, with minor QTLs located on BTA5, 9, 10, 11, 19, and 20. The validation population for pregnancy status at the end of the breeding season were Angus–Brahman crossbred heifers bred by natural mating. In concordance with the discovery population, the large QTL on BTA7 and QTLs on BTA10 and 12 were identified.

**Conclusions:**

In summary, QTLs and candidate SNPs identified were associated with pregnancy outcomes in beef heifers, including a large QTL associated with a group of protocadherin genes. Confirmation of these associations with larger populations could lead to the development of genomic predictions of reproductive function in beef cattle. Moreover, additional research is warranted to study the function of candidate genes associated with QTLs.

## Background

In beef cattle, more than 50% of the energy input to produce a unit of beef is consumed by the female that produced the calf [1]. Females that fail to get pregnant by the end of the breeding season are often culled from the herd because they consume valuable resources without contributing to the production system [2]. Moreover, females that become pregnant late in the breeding season have calves with lower weaning weights and longer subsequent calving intervals [3, 4]. Development of genomic tools to identify females with high genetic merit for reproductive function could increase the profitability and sustainability of beef production.

Fertility genetics is challenging because of the large number of physiological and environmental elements affecting reproductive success [5, 6]. Accordingly, heritability of fertility traits in beef cattle is low to moderate. For instance, a genomic analysis conducted with beef animals estimated the heritability of age at first calving to be 0.31 and of calving interval to be 0.02 [7]. In another study, heritability of these traits was 0.14 and 0.03, respectively [8]. Heritability estimates of heifer pregnancy rate by the end of breeding season ranged from 0.07 to 0.21 [9–11] and heritability for pregnancy to fixed-time artificial insemination (AI) ranged from 0.06 to 0.18 [10, 12]. Nonetheless, genetic progress of fertility traits is possible, as shown for daughter pregnancy rate in dairy cattle [13, 14]. Moreover, genomics can improve rates of genetic gain more for lowly-heritable traits than for highly-heritable traits [15].

Genome-wide association studies (GWAS) are a useful genomic analysis tool in livestock because it can form the basis for selection as well as delineate some of the biological causes for genetic differences among animals. There have been several studies reporting use of GWAS to identify quantitative trait loci (QTLs) for fertility traits of beef cattle. Among these studies are GWAS for age at first calving [16–18], pregnancy to AI after estrous synchronization [10] or after fixed-time AI [12], pregnancy outcome at the end of the breeding season [11], conception rate and number of services required for pregnancy [19, 20], rebreeding success after calving [21], and fertility classification following serial embryo transfers [22].

Here, GWAS were performed on two different measures of pregnancy outcomes in Angus–Brahman crossbred heifers bred by a combination of fixed-time AI and natural breeding. Objectives were to identify QTLs and candidate genes associated with the pregnancy traits and to perform a subsequent analysis on an unrelated population of heifers to verify these observations for one of the traits.

## Methods

### Discovery population

Data were collected on a population of Angus–Brahman crossbred heifers owned by the Seminole Tribe of Florida Inc., located in Northeast Glades, Florida, USA (27°04’ N 81°04’ W) (*n* = 2,272; average Brahman percentage = 23%; range 1% to 59%). Body weight at 4 months before breeding averaged 357 kg (range 229 to 528 kg). In November of each year of the study (2016–2018), heifers, approximately 2 years of age, were subjected to fixed-time AI using a 5-day Co-Synch + CIDR protocol. On d 0 of the protocol, heifers were administered 100 µg gonadotropin releasing hormone (GnRH) intramuscularly and a CIDR device containing 1.38 g progesterone (Eazi-Breed CIDR Cattle Insert; Zoetis Inc., Madison, NJ, USA) was inserted intravaginally. On d 5, the CIDR was removed and 25 mg prostaglandin F2α (PGF2α) was administered intramuscularly. Approximately 8 h after the first dose of PGF2α, another equal dose was administered. On d 8, immediately prior to AI, heifers received 100 µg GnRH intramuscularly (66 ± 2 h after CIDR removal). Each heifer was inseminated one time and then heifers were placed with bulls ~ 14 d later for natural mating for 90 d. Ultrasonic examination of the reproductive tract was conducted twice to determine pregnancy status at ~ 30 d after AI and again at the end of the breeding season.

### Validation population

Data were collected on a population of Angus–Brahman crossbred heifers owned by the Williamson Cattle Company of Okeechobee, Florida, USA (27°18’ N, 80°48’ W) (*n* = 325; average Brahman percentage = 32%; range 20% to 43%). Body weight of the heifers at 4 months before the start of breeding was 286 kg, with a range of 168 to 395 kg. Heifers, approximately 1 year of age, were placed with bulls for natural mating in November 2021. Each animal was classified as pregnant or not pregnant at the end of the 90-d breeding season after ultrasonic examination for presence of a fetus.

### GWAS

Genomic DNA was extracted from blood using the DNeasy Blood & Tissue kit (Qiagen, Valencia, CA, USA) according to manufacturer instructions and stored at −20 °C. Animals were genotyped with the Bovine GGP F-250 chip (GeneSeek, Lincoln, NE, USA) which contains 221,077 single nucleotide polymorphisms (SNPs). Position of SNPs were mapped using the ARS-UCD 1.2 *Bos taurus* sequence assembly. Quality control included the exclusion of non-autosomal SNP markers, minor allele frequency (MAF) of > 5%, a call rate of > 90% at the marker level, and a call rate of > 85% at the animal level.

In the discovery population, a total of 111,133 markers and 2,263 heifers remained after quality control. Two phenotypes were analyzed: pregnancy outcome to first-service AI (PAI; *n* = 1,481) and pregnancy status at the end of the breeding season (PEBS; *n* = 1,725). For both traits, a value of 0 was given to non-pregnant animals and 1 to pregnant animals.

In the validation population, a total of 107,249 markers and 319 heifers remained after quality control. The one phenotype available, pregnancy status at the end of the breeding season (*n* = 278), was analyzed.

Average information restricted maximum likelihood (AIREML) variance components and heritabilities for each trait were estimated using single-trait single-step genomic best linear unbiased prediction (ssGBLUP) from single-trait animal linear mixed models. Computations were performed using the *airemlf90* package from the BLUPF90 family of programs [23]. The single-trait animal mixed models included the direct additive genetic and residual as random effects; year of collection, body weight and percent Brahman were included as fixed effects. The single-trait animal mixed models were as follows:

$$\varvec{y}=\mathbf{X}\varvec{b}+\mathbf{Z}\varvec{u}+\varvec{e},$$

where ***y*** is a vector of phenotypic records, ***X*** is an incidence matrix linking phenotypic records to fixed effects, ***b*** is a vector of fixed effects, ***Z*** is an incidence matrix relating phenotypic records to direct additive genetic effects, ***u*** is a vector of random animal direct additive genetic effects, and ***e*** is a vector of random residuals. The random vectors ***u*** and ***e*** were distributed as $\varvec{u} \sim \text{N}(0,\varvec{G}{\sigma }_{u}^{2})$ and $\varvec{e} \sim \text{N}(0,\varvec{I}{\sigma }_{e}^{2})$, where ${\sigma }_{u}^{2}$ is the direct additive genetic variance, ${\sigma }_{e}^{2}$ is the residual variance, $\varvec{G}$ is the genomic relationship matrix, and $\varvec{I}$ is an identity matrix. The genomic relationship matrix was based on VanRaden [24], assuming allelic frequency from the population:

$$\varvec{G}= \frac{{\varvec{Z}\varvec{Z}}^{\varvec{{\prime }}}}{2{\Sigma }{p}_{i}(1-{p}_{i}) }$$

where ***Z*** is a centered incidence matrix of genotype covariates (0, 1, 2), and the denominator is a scaling parameter, where *p*_*i*_ is the frequency of the reference allele at the *i*-th SNP. The (co)variance matrix of ***u*** and ***e*** random vectors in single-trait models (${\varvec{V}}_{1}$) was as follows:

$${\varvec{V}}_{1}=\left[\begin{array}{cc}\varvec{G}{\sigma }_{u}^{2}& 0\\ 0& \varvec{I}{\sigma }_{e}^{2}\end{array}\right]$$

Genome wide associations were performed for each trait using the ssGBLUP procedure. The GWAS results are reported as the proportion of variance explained by a 200-kb window. Manhattan plots were produced using R software [25]. SNPs were mapped to genes using Ensembl version 107 [26]. Correlation between each of the phenotypic traits was analyzed using the Proc Corr procedure of SAS version 9.4 (SAS Institute, Cary, NC, USA).

### Functional analysis

A gene list was compiled using all genes associated with major QTLs identified for both traits in the discovery population. Ingenuity Pathway Analysis (IPA; Qiagen, Germantown, MD, USA) [27] was performed to identify physiological system development and function annotations as well as molecular and cellular function annotations overrepresented (*P* < 0.05) in the gene list associated with major QTLs.

## Results

### Summary of phenotypic traits in the discovery population

The average percent of females that were pregnant to fixed-time AI across all breeding seasons was 28.0%. The average percent of heifers pregnant at the end of the breeding seasons was 89.6%. The phenotypic correlation between PAI and PEBS was 0.194 (*P* < 0.0001).

### Pregnancy outcome to first-service AI

**Fig. 1:**
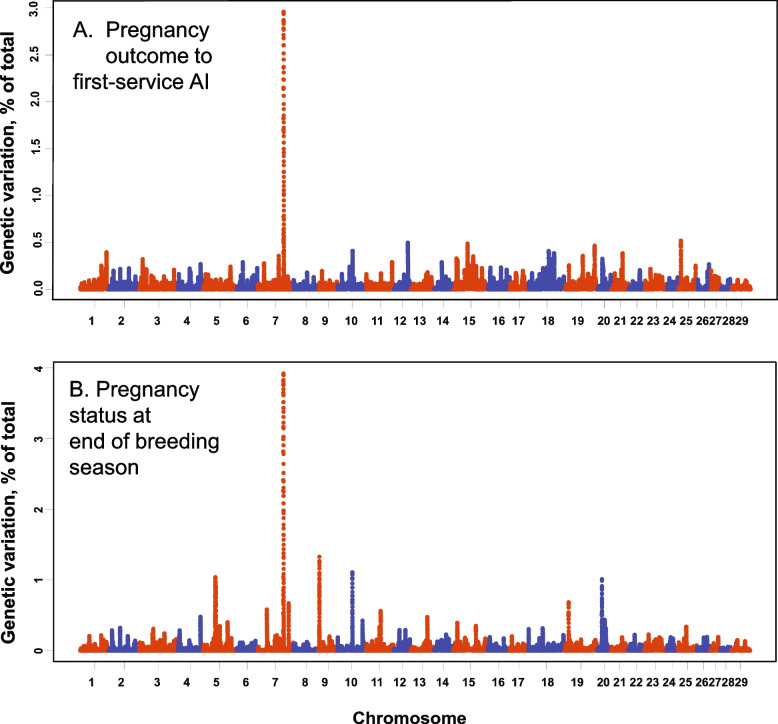
Manhattan plots displaying the association between genomic sliding windows for pregnant outcome to first-service AI (**A**) and pregnancy status at the end of the breeding season (**B**) in the discovery population

Heritability of PAI was estimated as 0.149, with a standard error of 0.053 (Table 1). The GWAS indicated a large QTL on *Bos taurus* autosome (BTA) 7 that explained almost 3% of the genetic variation (Fig. 1A). The QTL contained the genes *SLC25A2, PCDHGA3, PCDHGC3* and *PCDHGA5* within the 200 kb window. Other QTLs explaining roughly 0.5% of the variation each were identified on BTA12 and 25. A summary of all genes associated with these QTLs are in Table 2.

**Table 1:** Single-trait AIREML estimates of genetic variances (σ^2^_e_), residual variances (σ^2^_u_), and heritabilities (*h*^2^) with standard error (SE) for reproductive traits in the discovery population

Trait	σ^2^_e_	σ^2^_u_	*h*^2^ ± SE
Pregnancy outcome to first-service AI	0.03	0.17	0.149 ± 0.053
Pregnancy status at the end of the breeding season	0.01	0.08	0.122 ± 0.044

**Table 2:** Summary of the genes mapped to major QTL for pregnancy outcome to first-service AI and pregnancy status at the end of the breeding season in the discovery population

Trait	BTA	Position, Mb	Variance explained, %	Genes	
Pregnancy outcome to first-service AI	7	52.33–52.53	2.96	*PCDHGA3* *PCDHGA5*	*PCDHGC3* *SLC25A2*
	12	78.89–79.09	0.50	*BIVM* *ERCC5* *METTL21C*	*POGLUT2* *TEX30* *TPP2*
	25	2.63–2.83	0.52	*MEFV* *OR2C1* *OR1F1* *OR1F1C*	*OR1F1E* *ZNF75A* *ZNF200* *ZNF263*
Pregnancy status at the end of the breeding season	5	56.25–56.45	1.04	*GPR182* *LRP1* *MYO1A* *NAB2*	*NEMP1* *STAT6* *TAC3* *ZBTB39*
	7	13.13–13.33	0.58	-	-
	7	52.33–52.53	3.92	*PCDHGA3* *PCDHGA5*	*PCDHGC3* *SLC25A2*
	7	80.48–80.68	0.67	*ANKRD34B* *FAM151B* *FBLL1*	*RARS1* *WWC1* *ZFYVE16*
	9	10.61–10.81	1.33	-	-
	10	37.88–38.08	1.11	*CDAN1* *HAUS2*	*TTBK2*
	11	70.63–70.83	0.56	*ALK* *CLIP4*	*PCARE* *TOGARAM2*
	19	14.42–14.62	0.69	*CCL14* *CCL16* *CCL5* *C19H17orf50*	*HEATR9* *MMP28* *TAF15*
	20	21.99–22.19	1.01	*GPBP1*	

### Pregnancy status at the end of the breeding season

Heritability of PEBS was estimated as 0.122, with a standard error of 0.044 (Table 1). Several QTLs were identified by GWAS (Fig. 1B) including a QTL explaining almost 4% of the genetic variation on BTA7 that was synonymous with the QTL for PAI. Other, smaller QTLs were located on BTA5, 9, 10, 11, 19, and 20. Genes associated with major QTLs are described in Table 2.

**Fig. 2:**
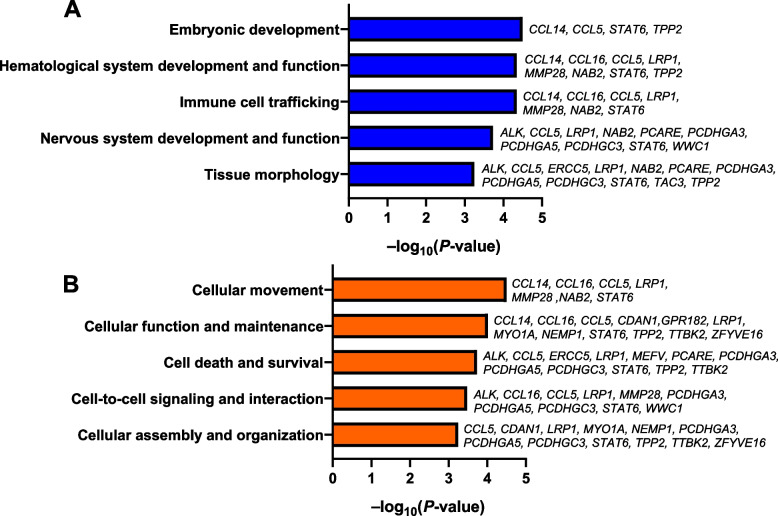
The top five physiological system and development annotations (**A**) and molecular and cellular functions (**B**) of the genes linked to major QTLs as determined by Ingenuity Pathway Analysis

IPA was used to identify physiological function annotations (Fig. 2A) and molecular and cellular functions (Fig. 2B) that were overrepresented for candidate genes (*P* < 0.05). The top physiological annotations were embryonic development, hematological system development and function, and immune cell trafficking. Cellular movement, cellular maintenance, and cellular development were the most significant molecular and cellular functions.

### Validation model

**Fig. 3:**
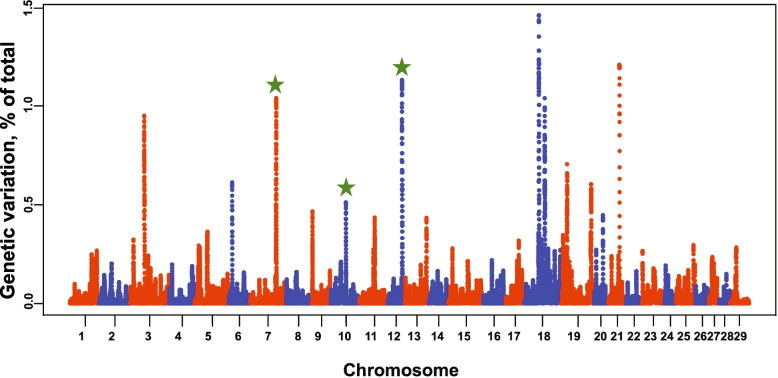
Manhattan plots displaying the association between genomic sliding windows for pregnancy status at the end of the breeding season in the validation population. QTLs that were also identified in the discovery population are marked with green stars

The average percent of heifers pregnant at the end of the breeding season was 93.9% in the Williamson population. GWAS results identified three QTLs on BTA7, 10, and 12 that were validated when compared to the original Seminole population (Fig. 3). The major QTL on BTA7 explained roughly 1% of the genetic variation. Other novel QTLs were located on BTA3, 6, 18, 19, and 21.

## Discussion

Quantitative trait loci, candidate genes and gene ontologies related to pregnancy phenotypes in the discovery population were identified using GWAS. Moreover, three QTLs for pregnancy status at the end of the breeding season were confirmed in a separate, smaller validation population. Among the specific genes linked to genetic variation in pregnancy outcomes were protocadherin genes and chemokine ligands as well as other genes such as *TAC3, STAT6, LRP1*, and *NAB2* that have been implicated in reproductive processes. Many of these candidate genes were overexpressed in functional annotations associated with reproductive processes such as embryonic development, hematological system function, immune function, and nervous system development and function.

The most striking result of the study was identification of a large QTL at BTA7 that explains the most genetic variation for both reproductive phenotypes. The identification of this QTL in the validation population suggests its potential importance for these fertility-related phenotypes. The QTL is located between 52.3 and 52.5 Mb and contains a linked group of γ-protocadherin genes, *PCDHGA3*, *PCDHGC3*, and *PCDHGA5*, as well as the inner mitochondrial membrane transporter *SLC25A2* that transports ornithine. Protocadherins, which are members of the cadherin superfamily, function in cell adhesion, signaling, and tissue morphogenesis [28]. Expression of *PCDHGC3* in bovine tissues has not been described but *PCDHGA3* and *PCDHGA5* are most highly expressed in neural tissues and the pituitary (http://cattlegeneatlas.roslin.edu.ac.uk). Mutations in the FAT/DCHS family of protocadherins have been associated with pituitary stalk developmental defects in the human [29]. Defects in anterior pituitary and infundibulum were identified in mice in which *Dchs1, Dchs2*, or *Cdhf14* were deleted [29]. Given the central importance for the hypothalamic-pituitary axis for control of reproductive processes including integration of nutritional cues [30, 31], it is possible that there are mutations in protocadherin genes that affect pituitary function in the bovine.

One candidate gene, *TAC3*, linked to QTL in BTA5 of the PEBS model has been implicated in regulation of nervous system or pituitary function. It encodes neurokinin B, which is critical in the control of GnRH secretion, an important mammalian reproductive regulator [32, 33]. Loss of function mutations of *TAC3* and its receptors have been associated with pubertal delays in humans [32]. *TAC3* was also differentially expressed in white blood cells between heifers pregnant to AI and those not pregnant to AI [34]. Other candidate genes identified here are also involved in regulation of transcription and cell signaling. *STAT6* is a member of the STAT transcription factor family. It was one of five transcription factors identified using network analysis as being associated with first-service conception in Brangus heifers [10] and was a predicted regulator of 452 differentially expressed genes associated with puberty in Brahman heifers [35]. *LRP1* is a member of the lipoprotein receptor family that encodes a protein that regulates signaling pathways like VEGF and TGF-β [36]. VEGF is vital for the development of angiogenesis throughout pregnancy at the embryonic and maternal interface, and polymorphisms in *VEGF* have been correlated to pregnancy complications and loss [37]. TGF-β in the endometrium has been linked with embryo receptivity and successful implantation in mice and other mammals [38]. Lastly, *NAB2* controls the transcription of *EGR1*, which is implicated in bovine ovarian fibrosis [39].

Another notable gene cluster associated with the QTL for PEBS on BTA19 included a series of chemokines, *CCL5*, *CCL14*, and *CCL16*, which regulate immune cells. Endometrial expression of these chemokines, specifically *CCL14* and *CCL16*, is increased during early pregnancy in the bovine and may have further functions relating to maternal recognition of pregnancy [40]. A role for these molecules in implantation and trophoblast migration have been identified in human as well [41]. The relationship between genes controlling immune function and reproductive outcomes is not surprising because one of the major determinants of whether cows become pregnant after calving is whether they experience an inflammatory disease [42].

There have been three other GWAS reported for analysis of traits similar to those analyzed here. Fortes et al. [10] evaluated conception rate at first service after estrous synchronization and for pregnancy rate at the end of the breeding season in Brangus heifers. Information of specific significant SNP identified by GWAS are not available but one of the transcription factors identified by gene network analysis as important for fertility was *STAT6* on BTA5, also associated with a large QTL in the current experiment study. Porto-Neto et al. [12] identified many SNPs associated with pregnancy outcome after fixed-time AI in heifers that were largely *Bos indicus* but there were no SNP analogous to the large QTL on BTA7 seen here. In a study with *Bos taurus*, Akanno et al. [11] examined pregnancy success in Canadian beef cattle at the end of the breeding season. There were only three significant SNPs associated with this trait on BTA9, BTA20 and BTA21.

Genome-wide association studies are often not repeatable [43] and it remains to be determined whether the QTL identified here will prove useful for predicting fertility in beef cattle. The fact that three QTLs were also identified in the validation population is an indication that the QTLs are predictive of fertility in more than one population; however, the validation population is limited in power due to the high pregnancy rate and subsequent low proportion of non-pregnant animals. Accumulation of more records from beef cattle populations may allow for further validation of these QTLs, which could subsequently lead to the development of accurate genomic estimates of reproductive function to improve selection of reproductive phenotypes in beef cattle. Another limitation of the study conducted here was that the traits of interest are binomially distributed but were analyzed as if they were linear. The error is considered small because results of genetic analysis of binomial data indicate high (> 0.9) correlations between linear and threshold models [44, 45].

## Conclusions

In summary, candidate QTLs associated with pregnancy outcomes in beef heifers were identified here, including one QTL on BTA7 explaining a large portion of genetic variation. Three QTLs were then confirmed in a second population. Confirmation of these associations with larger populations could lead to the development of genomic estimates of reproductive function in beef cattle. Moreover, additional research is warranted to study the function of candidate genes associated with these QTLs.

## Data Availability

Genomic data are available through the European Variation Archive (EVA), accession number PRJEB60100. Phenotypic data are available through Dryad, doi: 10.5061/dryad.h70rxwdp5.

## Abbreviations

AIREML: Average information restricted maximum likelihood
BTA: *Bos taurus* autosome
CCL5: C-C motif chemokine ligand 5
CCL14: C-C motif chemokine ligand 14
CCL16: C-C motif chemokine ligand 16
CDHF14: FAT atypical cadherin 4
CIDR: Controlled intravaginal drug release
DAVID: Database for annotation, visualization, and integrated discovery
DCHS1: Dachsous cadherin-related 1
DCHS2: Dachsous cadherin-related 2
EGR1: Early growth response 1
GnRH: Gonadotropin releasing hormone
GWAS: Genome-wide association study
IPA: Integrated pathway analysis
LRP1: LDL receptor related protein 1
MAF: Minor allele frequency
NAB2: NGFI-A binding protein 2
PAI: Pregnancy outcome to first-service artificial insemination
PCDHGA3: Protocadherin gamma subfamily A, 3
PCDHGA5: Protocadherin gamma subfamily A, 5
PCDHGC3: Protocadherin gamma subfamily C, 3
PEBS: Pregnancy status at the end of the breeding season
PGF2α: Prostaglandin 2-alpha
QTL: Quantitative trait locus
SLC25A2: Solute carrier family 25 member 2
SNP: Single nucleotide polymorphism
ssGBLUP: Single-step genomic best linear unbiased prediction
STAT6: Signal transducer and activator of transcription 6
TAC3: Tachykinin precursor 3
TGF-β: Transforming growth factor beta 1
VEGF: Vascular endothelial growth factor
